# MYCT1 inhibits hematopoiesis in diffuse large B-cell lymphoma by suppressing RUNX1 transcription

**DOI:** 10.1186/s11658-023-00522-0

**Published:** 2024-01-03

**Authors:** Ying Liang, Xin Wei, Peng-Jie Yue, He-Cheng Zhang, Zhen-Ning Li, Xiao-Xue Wang, Yuan-Yuan Sun, Wei-Neng Fu

**Affiliations:** 1 Department of Medical Genetics, China Medical University, No. 77 Puhe Road, Shenyang North New Area, Shenyang, Liaoning Province 110122 People’s Republic of China; 2 Department of Hematology, The First Affiliated Hospital of China Medical University, Shenyang, 110001 People’s Republic of China; 3 Department of Oromaxillofacial-Head and Neck Surgery, Liaoning Province Key Laboratory of Oral Disease, School and Hospital of Stomatology, China Medical University, Shenyang, People’s Republic of China

**Keywords:** MYCT1, Diffuse large B-cell lymphoma, Chromosomal karyotype

## Abstract

**Background:**

The abnormality of chromosomal karyotype is one factor causing poor prognosis of lymphoma. In the analysis of abnormal karyotype of lymphoma patients, three smallest overlap regions were found, in which MYCT1 was located. MYCT1 is the first tumor suppressor gene cloned by our research team, but its studies relating to the occurrence and development of lymphoma have not been reported.

**Methods:**

R banding analyses were employed to screen the abnormality of chromosomal karyotype in clinical specimen and MYCT1 over-expression cell lines. FISH was to monitor MYCT1 copy number aberration. RT-PCR and Western blot were to detect the mRNA and protein levels of the MYCT1 and RUNX1 genes, respectively. The MYCT1 and RUNX1 protein levels in clinical specimen were evaluated by immunohistochemical DAB staining. The interaction between MYCT1 and MAX proteins was identified via Co-IP and IF. The binding of MAX on the promoter of the RUNX1 gene was detected by ChIP and Dual-luciferase reporter assay, respectively. Flow cytometry and CCK-8 assay were to explore the effects of MYCT1 and RUNX1 on the cell cycle and proliferation, respectively.

**Results:**

MYCT1 was located in one of three smallest overlap regions of diffuse large B-cell lymphoma, it altered chromosomal instability of diffuse large B-cell lymphoma cells. MYCT1 negatively correlated with RUNX1 in lymphoma tissues of the patients. MAX directly promoted the RUNX1 gene transcription by binding to its promoter region. MYCT1 may represses RUNX1 transcription by binding MAX in diffuse large B-cell lymphoma cells. MYCT1 binding to MAX probably suppressed RUNX1 transcription, leading to the inhibition of proliferation and cell cycle of the diffuse large B-cell lymphoma cells.

**Conclusion:**

This study finds that there is a MYCT1-MAX-RUNX1 signaling pathway in diffuse large B-cell lymphoma. And the study provides clues and basis for the in-depth studies of MYCT1 in the diagnosis, treatment and prognosis of lymphoma.

**Supplementary Information:**

The online version contains supplementary material available at 10.1186/s11658-023-00522-0.

## Background

Lymphoma is a malignant tumor caused by clonal proliferation of lymphoid-derived cells. According to histopathological classification, lymphoma is divided into two types, Hodgkin lymphoma (HL) and non-Hodgkin lymphoma (NHL) [1]. Diffuse large B-cell lymphoma (DLBCL) is the most common subtype of NHL. DLBCL is invasive and often infiltrates the bone marrow, indicating that it has a high degree of malignancy, rapid progression and poor prognosis [2]. In recent years, the survival rate of patients has risen greatly, but 40% of patients will deteriorate to recurrent and refractory DLBCL (R/R), leading to a mortality rate of up to 23.3%, seriously threatening human health [3]. DLBCL involves a large number of gene mutations, copy number variations and chromosomal karyotype abnormalities [4]. Studying the mechanism of MYCT1 in the occurrence and development of DLBCL at the levels of cytogenetics and molecular genetics is helpful to the identification of molecular targets for the diagnosis, treatment and prognostic prediction of DLBCL.

Chromosomal instability (CIN) is an important trait of tumor cells and is characterized by abnormal numbers and structures. CIN is commonly found in a variety of solid tumors and malignant hematological diseases [5, 6]. Studies have shown that specific marker chromosomes have become pivotal targets for the diagnosis, treatment and prognostic prediction of chronic myeloid leukemia and other tumors [7].

*MYCT1* (MYC target 1), located on chromosome 6q25, was first discovered and cloned by our research group in laryngeal cancer and was once named the *C-MYC target from laryngeal cancer cells* (*MTLC*) [8]. Studies have shown that MYCT1 plays different roles in different tumors, suggesting that it has tissue specificity [9–15]. However, the role and mechanism of MYCT1 in lymphoma, including DLBCL, have not been reported.

In a previous study, we found several differentially expressed genes related to the occurrence and development of lymphogenic malignant diseases in laryngeal cancer cells stably transformed with *MYCT1*, including RUNX1 (runt related protein 1). RUNX1 is an important transcription factor [16]. Similar to *MYCT1*, *RUNX1* shows two-sided roles in different tumors. Even in lymphoma, its role is controversial based on studies from different groups.

Moreover, we also found that *MYCT1 and RUNX1* were downregulated and upregulated in lymphoma, respectively. Meanwhile, deletion of the *MYCT1* locus and amplification of the *RUNX1* locus occurred in lymphoma cell karyotypes, suggesting that MYCT1 and RUNX1 have opposite roles in lymphoma.

Previously, we discovered that MYCT1 interacted with MAX (MYC associated Factor X, MAX) in laryngeal carcinoma. Bioinformatic prediction revealed that there was a potential binding site of MAX in the *RUNX1* promoter [10]. Since MYCT1 and MAX are widely expressed in a variety of normal tissues, we speculate that MYCT1 may regulate the expression of RUNX1 at the transcriptional level through interaction with MAX, participating in the occurrence and development of lymphoma.

In this study, we examine the effects of MYCT1 on the karyotype and proliferation of lymphoma cells by using cytogenetic and molecular genetic techniques and explore the related molecular mechanisms of MYCT1 in lymphoma to provide important clues for the in-depth study of MYCT1-related pathways in the diagnosis, treatment and prognostic prediction of lymphoma.

## Methods

### Clinical specimen collection

Bone marrow karyotype samples of 209 lymphoma patients with bone marrow infiltration provided by the Department of Hematology of the First Affiliated Hospital of China Medical University from 2012 to 2022 were collected. Lymphoma patients were diagnosed with malignant lymphoma with bone marrow infiltration according to WHO diagnostic criteria, all of whom were in clinical stage IV.

From 2017 to 2022, 27 paraffin sections of newly diagnosed lymphoma patients and 27 patients with reactive lymphadenitis were collected from the pathology department of the First Affiliated Hospital of China Medical University([2022] 2022-377-2, Oct 12, 2022). The pathological diagnosis of lymphoma and reactive lymphadenitis in all patients was confirmed by pathological experts.

### Cell culture and construction of stable MYCT1 over-expression cell lines

The human diffuse large B-cell lymphoma cell lines DB and SU-DHL4 were purchased from ATCC in the United States.

All cells were cultured in RPMI 1640 supplemented with 10% fetal bovine serum, 100 U/mL penicillin and 100 μg/mL streptomycin. After culturing in a 37 °C 5% CO2 incubator to the third generation, the cells were used for subsequent experimental research.

The cells in logarithmic growth phase were divided into 3 × 10^5^/well, inoculated in a six-well plate, and then added to the over-expression MYCT1 plasmid and transfection mixture incubated at room temperature. The solution was mixed gently and continued to culture. After 48 h, the medium was replaced with fresh medium to continue the culture or carry out the next experiment.

### Analysis of chromosome aberrations in clinical patients and stable MYCT1 overexpression cell lines

Bone marrow cells were extracted from the patient, and 3 × 10^6^ cells were inoculated into bone marrow cell culture medium. After culture at 37 °C for 18 h, 80 μL and 160 μL colchicine were added at 37 °C for 1 h. The cells were blocked in metaphase, 0.0075 mmol/L KCl hypotonic solution (6–8 mL preheated to 37 °C) was added for 38 min, and fixing solution was added successively for prefixation and fixation. The cell suspension was dropped onto a pretreated glass slide, placed into the band display solution heated to a constant temperature of 87.5 °C, and then subjected to Giemsa staining. The karyotypes were analyzed and counted under a microscope. The karyotype abnormalities were described according to ISCN 2020.

### Fluorescence in situ hybridization (FISH) analysis in clinical patients

The clinical patient cell suspension was dropped onto the slide. The slide was placed in 2 × SSC for 5 min, followed by 70%, 85% and 100% alcohol gradient dehydration for 2 min. After drying, 10 μL of probe suspension was added, and the slide was denatured at 88 °C for 2 min and hybridized at 45 °C for 2 h. The slide was washed in hybridization washing solution at 68 °C for 2 min and ddH_2_O at 37 °C for 1 min. After drying, 10 μL DAPI was added, and the results were observed by fluorescence microscopy. Two hundred split images were analyzed for each specimen, and the signal mode was described and sorted according to ISCN 2020.

### Quantitative real-time PCR analysis

Bone marrow samples were extracted with lymphocyte separation solution. Then, the red blood cells were broken, and RNA was extracted routinely. Pathological paraffin section specimens were analyzed according to a paraffin embedded tissue RNA Extraction Kit (Beijing, Tiangen Biotech). Reverse transcription of mRNA was performed with PrimeScript™ RT Master Mix (Takara). Quantitative real-time PCR was performed with SYBR® PremixExTaq™ II (Takara) in an ABI 7500 (Thermo). GAPDH was used as an endogenous control for normalization of mRNA expression, and fold change values were calculated by the 2^−ΔΔCt^ method.

### Western blot for the detection of MYCT1 and RUNX1

After the samples were washed with PBS, RIPA lysis buffer was added, and the samples were placed on ice for 30 min and centrifuged at 4 °C and 14,000 r/min for 20 min. The upper protein solution was aspirated into a new EP tube. The protein concentration was determined by a BCA protein analysis kit (Beyotime). The proteins were added to the solution for SDS‒PAGE at 80 V for 30 min, and then the voltage was adjusted to 120 V for 1 h. Proteins were transferred to PVDF membranes at 200 mA for 1 h. Then, the samples were washed with PBST. The PVDF membrane was oscillated and sealed with sealing solution for 2 h. After the samples were further washed with PBST, the MYCT1, RUNX1 and GAPDH antibody working solutions were added and hybridized overnight on a horizontal shaking table at 4 °C. After another wash with PBST, the secondary antibody was added and incubated on a shaking table for 2 h. After further washing, the prepared electrochemiluminescence kit (Tanon) was added, and after the sample was scanned in a ChemiDoc™ Touch Imaging System, the relative integrated density was analyzed.

### Flow cytometry to detect the cell cycle in BD and SU-DHL4 cells

The cells were collected in a 15 mL centrifuge tube and centrifuged at 800 r/min for 5 min. After the supernatant was discarded, the precipitate was washed with PBS and centrifuged for 5 min at 800 r/min. 500 μL of 70% ethanol was to the cell precipitate. After the cells were mixed, they were placed into a refrigerator at − 20 °C for 2 h. After fixation, the samples were centrifuged at 800 r/min for 5 min. PI working solution was added to the cell precipitate. After blowing and mixing, the mixture was kept away from light at room temperature for 30 min. The fluorescence was detected at 488 nm by flow cytometry.

### CCK-8 assay to detect cell proliferation after MYCT1 and RUNX1 over-expression

The cells to be tested were collected in a 15 mL centrifuge tube and centrifuged at 800 r/min for 5 min. The supernatant was discarded, and the final cell concentration was adjusted to 1 × 10^5^ with the culture medium. The cells in each test group and the control group needed 5 replicate wells, with 100 μL of cell suspension per well, cultured in an incubator for 24 h, 48 h, 72 h and 96 h. CCK8 reagent (10 μL/well) was added, bubbles were avoided, and incubation was continued at 37 °C for 2 h. The absorption light was detected by an enzyme labeling instrument at a wavelength of 490 nm.

### Coimmunoprecipitation (Co-IP)

After the collected cells were lysed, they were centrifuged at 14,000 *g* at 4 °C for 15 min. The supernatant was collected into new EP tubes, and 40 μL cleaved protein was absorbed from each EP tube reserved for input. Fifty microliters of pretreated agarose beads and 1 µL IgG antibody were added to the two tubes of remaining protein, which were then rotated at 4 °C for 2 h. After centrifugation, the target antibody and IgG (1 μL) were added to the supernatant, and the tubes were rotated at 4 °C overnight. Pretreated agarose beads (50 mL/tube) were added again, with further at 4 °C for 4 h. The supernatant was discarded after low-speed centrifugation, and the agarose beads were gently washed with PBS. The supernatant was discarded after centrifugation at 1000 r/min for 1 min, and 2 × loading of the same volume as the residual solution was added. After instantaneous centrifugation, the supernatant was taken for immunoblotting.

### Immunohistochemistry (IHC)

The slices were fixed in a sequence gradient and washed with distilled water after dehydration. After antigen repair, the samples were shaken and washed after 8 min of medium fire, boiling, cease-fire and low fire in a microwave oven; incubated with hydrogen peroxide working solution for 25 min; washed with pH 7.4 PBS; then, 3% BSA was added, and the solution was incubated for 30 min. Primary antibodies (MYCT1 1/100, RUNX1 1/100) were added and incubated overnight at 4 °C. After PBS washing at pH 7.4, the secondary antibody was incubated for 60 min. DAB color development was performed after another wash with PBS. Hematoxylin stain was added in turn, and neutral gum was used to seal the solution after gradient dehydration. Microscopic examination, image acquisition and analysis were subsequently performed.

### Chromatin immunoprecipitation (ChIP)

Next, 37% formaldehyde was added to the cell suspension for the cross-linking reaction, and 10 × glycine was added to eliminate formaldehyde. The mixture was centrifuged at 1200 r/min for 5 min, and the supernatant was discarded. ChIP was performed according to the manufacturer's instructions for the EZ-Magna ChIP™ kit (Millipore, Billerica MA, USA). A 1% agarose gel was prepared, electrophoresis of PCR products was performed, and ChemiDoc™ imaging was performed after ethidium bromide staining. The touch imaging system (Bio-Rad) was used to collect images and analyze gray values.

### Dual-luciferase reporter assay

A 24-well plate was selected, and the final concentration of 293 T cells was adjusted to 1 × 10^6^. The culture medium was added to a constant volume of 100 mL. After 24 h, the culture medium was discarded, PBS was added, and the cells were centrifuged to remove PBS. PLB lysate (100 mL) was added to the wells and shaken on a shaking table at room temperature for 20 min. Flocculent sediment was observed. After blowing, mixing and precipitation, 20 μL solution was added to a 96-well white special board. LAR II solution (100 μL) was added to the bottom of the hole, placed into the instrument, and measured immediately, and the luminous value was recorded. Then, 100 μL of termination solution was placed into the instrument, and the luminous value was measured immediately. Sea kidney was used as the internal reference for statistical analysis.

### Statistical analysis

GraphPad Prism 7.0 and SPSS 21.0 were used for statistical analysis. The Kolmogorov‒Smirnov test verifies a normal distribution. Continuous variables are expressed as the mean ± standard deviation. The unique *t* test was used for difference analysis, and the nonparametric rank sum test was used for difference analysis for data with a nonnormal distribution. The rate or composition of categorical variables was determined with the Chi square est. Kaplan‒Meier curves were used for survival analysis. All tests were bilateral tests, and *P* < 0.05 was considered to indicate statistical significance.

## Results

### MYCT1 is located in one of the three smallest overlapping regions of diffuse large B-cell lymphoma

**Fig. 1:**
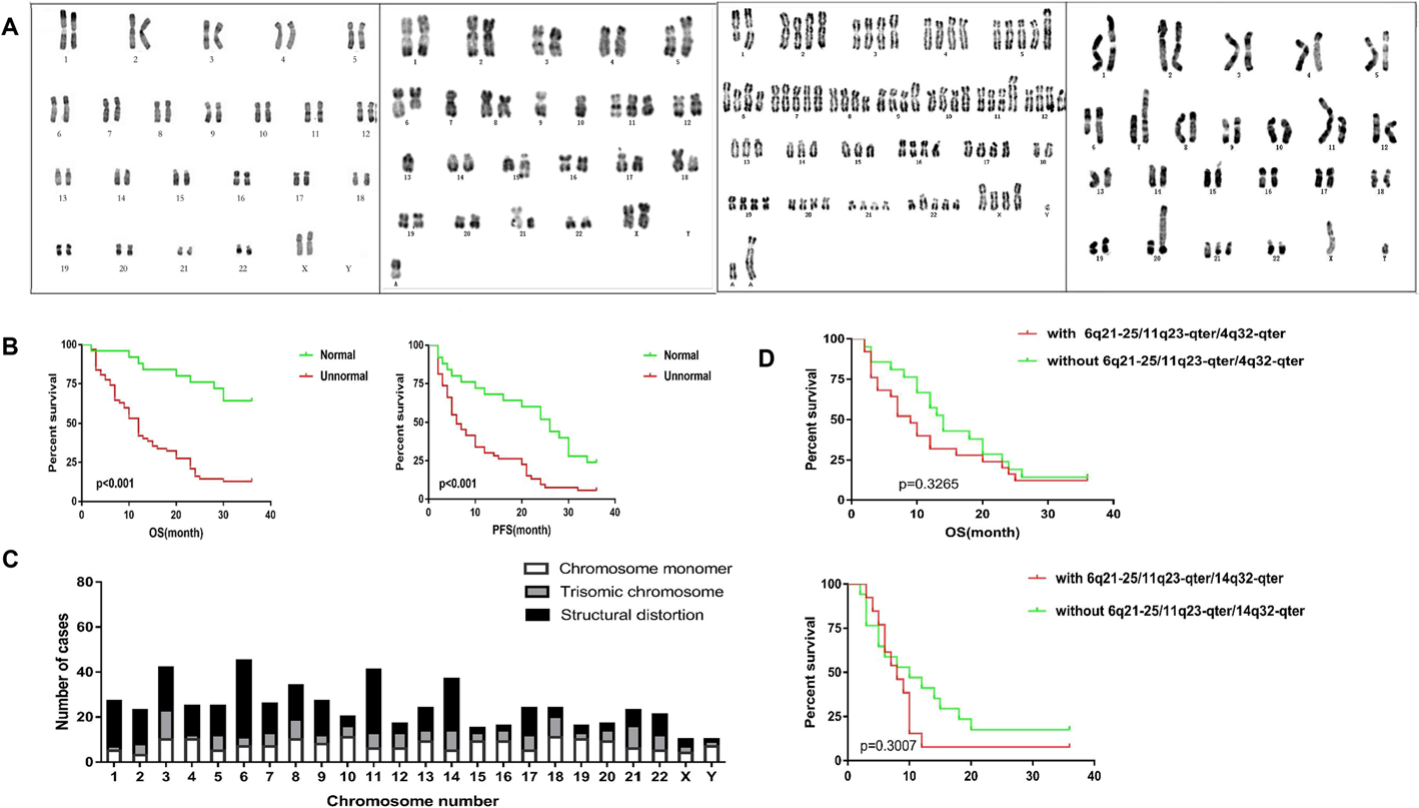
Karyotype analysis results and efficacy evaluation of patients with bone marrow infiltrating lymphoma. **A** Representative chromosome karyotype analysis results. The picture on the left shows a normal karyotype. The three figures on the right show the representative subdiploid karyotype, near tetraploid karyotype and hyperdiploid karyotype of lymphoma patients. **B** Statistical analysis results of chromosome karyotype, OS and PFS. **C** Overall situation of chromosome karyotype number and structural aberration in patients with abnormal karyotypes. **D** Effects of the three smallest overlapping regions on the OS and PFS of patients

We analyzed 209 lymphoma patients with bone marrow infiltration; 131 cases were normal, and 78 cases were abnormal in karyotype (Fig. 1A). We found that the platelet count and complete remission rate of patients with abnormal karyotypes were significantly lower than those of patients with normal karyotypes (*p* < 0.05), and the 3-year mortality rate was significantly higher than that of patients with normal karyotypes (*p* < 0.05) (Table 1). We analyzed the karyotype, overall survival (OS) and progression-free survival (PFS) of patients after routine combined chemotherapy. The results showed that the average OS and PFS of patients with abnormal karyotypes were significantly lower than those of patients with normal karyotypes (*p* < 0.05) (Fig. 1b), suggesting that patients with abnormal karyotypes had a short survival time, which was consistent with the high 3-year mortality.

**Table 1:** Karyotype and clinical data in patients with bone marrow infiltrating lymphoma

	Chromosome karyotype		*P*
	Normal	Abnormal	
Female/Male, *n*	54/77	22/56	0.058
Age < 60/ ≥ 60, *n*	67/64	32/46	0.156
Median WBC (range) (*109/L)	6.72 (2.8–253)	9.61 (0.66–475)	0.1027
Median Hb (range) (g/L)	112 (76–167)	113 (60–159)	0.9444
Median PLT(range)( *109/L)	146.5 (11–386)	94 (9–316)	0.0066
Lymphoma cells (range) (%)	34.8 (2.4–99.2)	46.7 (3–99)	0.2435
Complete remission, *n* (%)	68.7	30.7	0.001
Die during 3 year, *n* (%)	43.5	65.4	0.002

Among 78 patients with abnormal karyotypes, 68 (87.2%) showed complex karyotypes, in which three or more chromosomes were involved. A total of 589 chromosomal aberrations occurred, of which 317 (53.8%) were numerical aberrations and 272 (46.2%) were structural aberrations. The overall involvement rates of chromosomes 3, 6 and 11 were the highest, at 45 (7.65%), 43 (7.31%) and 41 (6.97%), respectively. Among the 317 chromosome number aberrations, 177 chromosome deletions (55.8%) and 140 chromosome expansions (44.2%) occurred. Trisomics and monomers were common. The most common trisomics were + 3 (13 cases, 4.1%), + 21 (10 cases, 3.2%), + 8 (9 cases, 2.8%), + 14 (9 cases, 2.8%), and + 18 (9 cases, 2.8%). The most common monomers were − 10 (11 cases, 3.5%), − 18 (11 cases, 3.5%), − 3 (10 cases, 3.2%), − 4 (10 cases, 3.2%), − 8 (10 cases, 3.2%), and − 19 (10 cases, 3.2%). Among the structural aberrations, chromosome 6 (34 cases, 12.5%), chromosome 11 (28 cases, 10.3%) and chromosome 14 (23 cases, 8.5%) were most commonly involved (Fig. 1c, Additional file 1: Table S1).

The smallest overlapping regions involved in structural abnormalities were 14q32-qter (18 cases, 6.6%), 6q21-25 (16 cases, 5.9%), and 11q23-qter (13 cases, 4.8%) (Table 2). We also analyzed the effects of the three smallest overlapping regions on the OS and PFS of patients. The results showed that there was no statistically significant difference in OS and PFS between the patients with the three smallest overlap areas and those with other karyotype abnormalities (*p* = 0.3265, *p* = 0.3007). However, the median OS and median PFS of patients with the three smallest overlap regions were 9 months and 8 months, respectively, and those with other abnormal karyotypes were 14 months and 10 months, respectively. The median OS and PFS of patients with the three smallest overlap regions were lower than those of patients with other abnormal karyotypes (Fig. 1D), suggesting that patients with abnormal smallest overlap regions may die earlier and have shorter progression-free survival. Since MYCT1 is located in one of the smallest overlapping regions, 6q21-25, follow-up studies focused on MYCT1.

**Table 2:** Chromosome structural aberrations in patients with abnormal karyotypes

Chromosome	SOR	Cases (%)	Chromosome	SOR	Cases (%)
X	Xq25-qter	2 (0.74%)	9	9p12-13	2 (0.74%)
Y	Yp11.3-pter	1 (0.37%)		9q13-22	9 (3.31%)
1	1p36-pter	3 (1.10%)		9q34-qter	2 (0.74%)
	1p13-32	4 (1.47%)	10	10p15-pter	2 (0.74%)
	1q32-44	7 (2.57%)	11	11p15-pter	3 (1.10%)
2	2q32-33	4 (1.47%)		11q14-22	2 (0.74%)
	2q36-qter	6 (2.21%)		11q23-qter	13 (4.78%)
3	3p21	4 (1.47%)	12	12p12-pter	4 (1.47%)
	3q25	5 (1.84%)	13	13q14-22	4 (1.47%)
	3q29-qter	5 (1.84%)		13q34-qter	4 (1.47%)
4	4p14-16	4 (1.47%)	14	14p11-pter	4 (1.47%)
	4q25	3 (1.10%)		14q32-qter	18 (6.62%)
	4q35-qter	3 (1.10%)	15	15q22	3 (1.10%)
5	5p14-15	6 (2.21%)		15q26-qter	7 (2.57%)
	5q13-33	2 (0.74%)	16	16q24-qter	2 (0.74%)
	5q35-qter	3 (1.10%)	17	17p12-13	5 (1.84%)
6	6p25-pter	5 (1.84%)		17q21	3 (1.10%)
	6q13	3 (1.10%)		17q25-qter	2 (0.74%)
	6q21-25	16 (5.88%)	18	18p11-pter	2 (0.74%)
	6q27-qter	7 (2.57%)		18q23-qter	2 (0.74%)
7	7p13-15	2 (0.74%)	19	19p13-pter	2 (0.74%)
	7p22	6 (2.21%)	20	20p13	3 (1.10%)
	7q22-24	5 (1.84%)	21	21p11-pter	3 (1.10%)
8	8p22-23	6 (2.21%)		21q21-qter	3 (1.10%)
	8q24	8 (2.94%)	22	22q13-qter	4 (1.47%)

### MYCT1 alters the chromosomal instability of diffuse large B-cell lymphoma cells

**Fig. 2:**
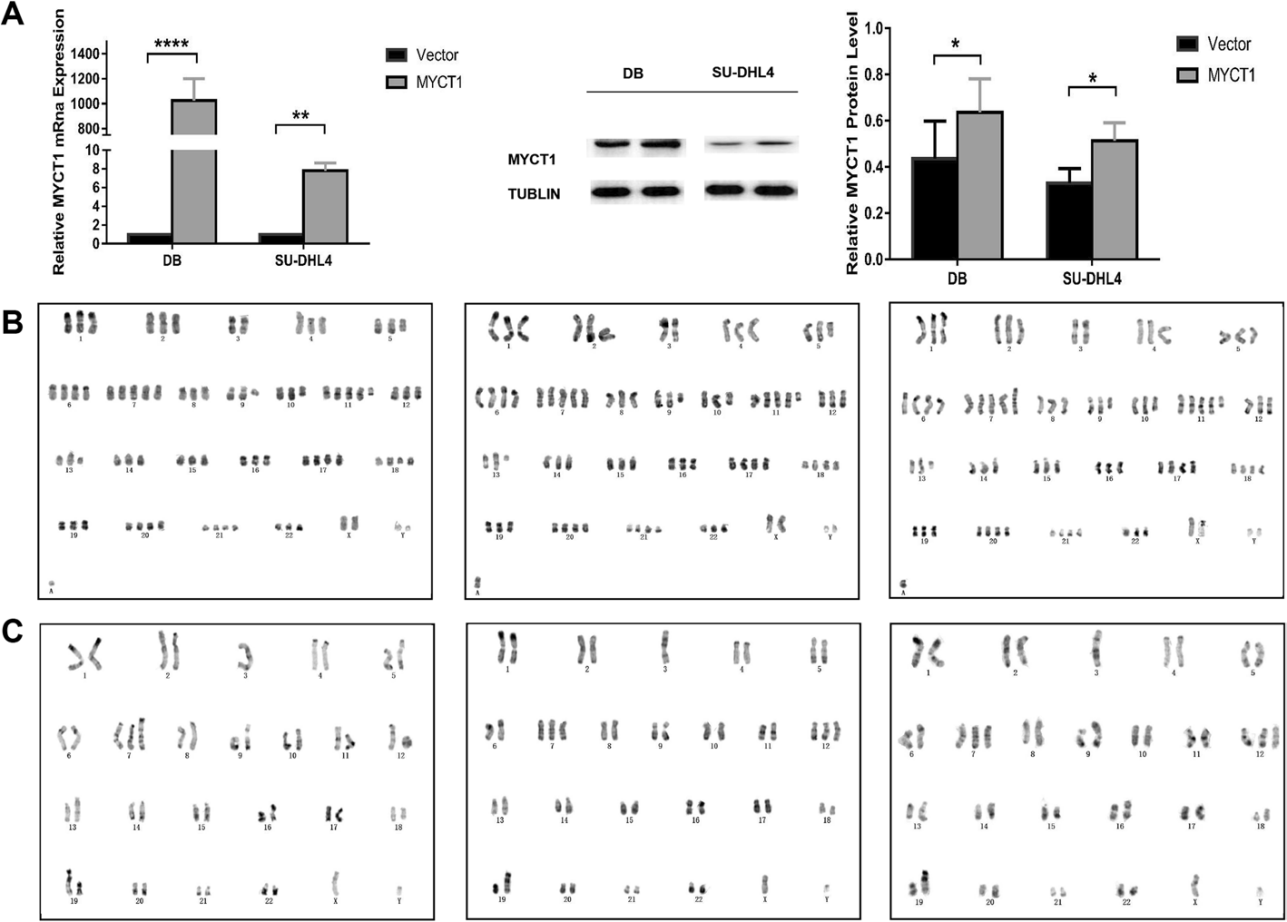
Effect of MYCT1 on the karyotypes of DLBCL cell lines DB and SU-DHL4. **A** Establishment of the DLBCL cell lines DB and SU-DHL4 with MYCT1 overexpression. The left figure shows the expression level of MYCT1 mRNA in DLBCL cells after stable transformation of the MYCT1 overexpression vector. The right figure shows the expression level of MYCT1 protein in DLBCL cells after stable transformation of the MYCT1 overexpression vector. *, * * and * * * * are *p* < 0.05, *p* < 0.01 and *p* < 0.0001, respectively. **B** Effect of MYCT1 on the karyotype of the DB cell line. The left picture shows the DB control, the middle picture shows the DB vector, and the right picture shows DB-MYCT1. **C** Effect of MYCT1 on the karyotype of the SU-DHL4 cell line. The left picture shows the SU-DHL4 control, the middle picture shows the SU-DHL4 vector, and the right picture shows SU-DHL4-MYCT1

Lentiviral vectors overexpressing MYCT1 were transfected into DB and SU-DHL4 cell lines. The mRNA and protein expression levels of MYCT1 after transfection were significantly higher than those of the control group (*p* < 0.05), suggesting that DB and SU-DHL4 cells with stable MYCT1 overexpression were successfully constructed (Fig. 2A).

This study analyzed the karyotypes of DB and SU-DHL4 cells after MYCT1 overexpression, and the results showed that no obvious abnormalities were found in the karyotypes of the empty group and control group. Chromosome changes were found in the karyotypes of DB and SU-DHL4 cells in the MYCT1 overexpression group. Structural abnormalities occurred in the DB cell line: a balanced translocation occurred between the short arm of chromosome 2 and the long arm of chromosome 8, and an equibrachial abnormal chromosome of the long arm of chromosome 7 was added at the same time. The short arms of chromosomes 18, 22 and 9 of the SU-DHL4 cell line all increased chromosome fragments of unknown origin, and a long arm of chromosome 7 was added. The above karyotype results suggest that MYCT1 could cause changes in chromosome instability, resulting in changes in the number and structure of chromosomes (Fig. 2B, C, Table 3).

**Table 3:** Karyotypic differences in DB and SU-DHL4 cell lines by MYCT1

Cell line	Chromosome mode	Chromosomal structural changes	Differential structural changes
DB Control	79	+ Y,-3,add(4)(p16),del(5)(q31q33), + del(6)(p22), + 7, + 7,del(9)(q12q22),del(10)(q23), + 11, + del(11)(q13),der(13)t(8;13)(q22;q34),del(13)(q13q33),t(14;18)(q32;q21), + 17, + 18, + 20, + 21, + mar	
DB-Vector	79	+ Y,-3,add(4)(p16),del(5)(q31q33), + del(6)(p22), + 7, + 7,del(9)(q12q22),del(10)(q23), + 11, + del(11)(q13),der(13)t(8;13)(q22;q34),del(13)(q13q33),t(14;18)(q32;q21), + 17, + 18, + 20, + 21, + mar	
DB-MYCT1	79	+ Y,t(2;8)(p23;q24),-3,add(4)(p16),del(5)(q31q33), + del(6)(p22), + 7, + i(7)(q11),del(9)(q12q22),del(10)(q23), + 11, + del(11)(q13),der(13)t(8;13)(q22;q34),del(13)(q13q33),t(14;18)(q32;q21), + 17, + 18, + 20, + 21, + mar	t(2;8)(p23;q24) + i(7)(q11)
SU-DHL4 Control	46, 47	XY,t(2;8)(p23;q24),-3,der(3)t(3;11)(p26;q11), + 7, + der(7)t(7;12)(p11;q11), + 12,der(12),der(13)t(8;13)(q22;q34),t(14;18)(q32;q21),der(19)t(7;19)(q11;q13)	
SU-DHL4- Vector	46, 47	XY,t(2;8)(p23;q24),-3,der(3)t(3;11)(p26;q11), + 7, + der(7)t(7;12)(p11;q11), + 12,der(12),der(13), t(8;13)(q22;q34),t(14;18)(q32;q21),der(19)t(7;19)(q11;q13)	
SU-DHL4- MYCT1	46, 47	XY,t(2;8)(p23;q24),-3,der(3)t(3;11)(p26;q11), + der(7)t(7;12)(p11;q11), + del(7)(p11),add(9)(p24), + 12,der(12),der(13)t(8;13)(q22;q34),t(14;18)(q32;q21),add(18)(p11),der(19)t(7;19)(q11;q13),add(22)(p11)	+ del(7)(p11) add(18)(p11) add(22)(p11) add(9)(p24)

### MYCT1 represses RUNX1 transcription by binding MAX in diffuse large B-cell lymphoma cells

**Fig. 3:**
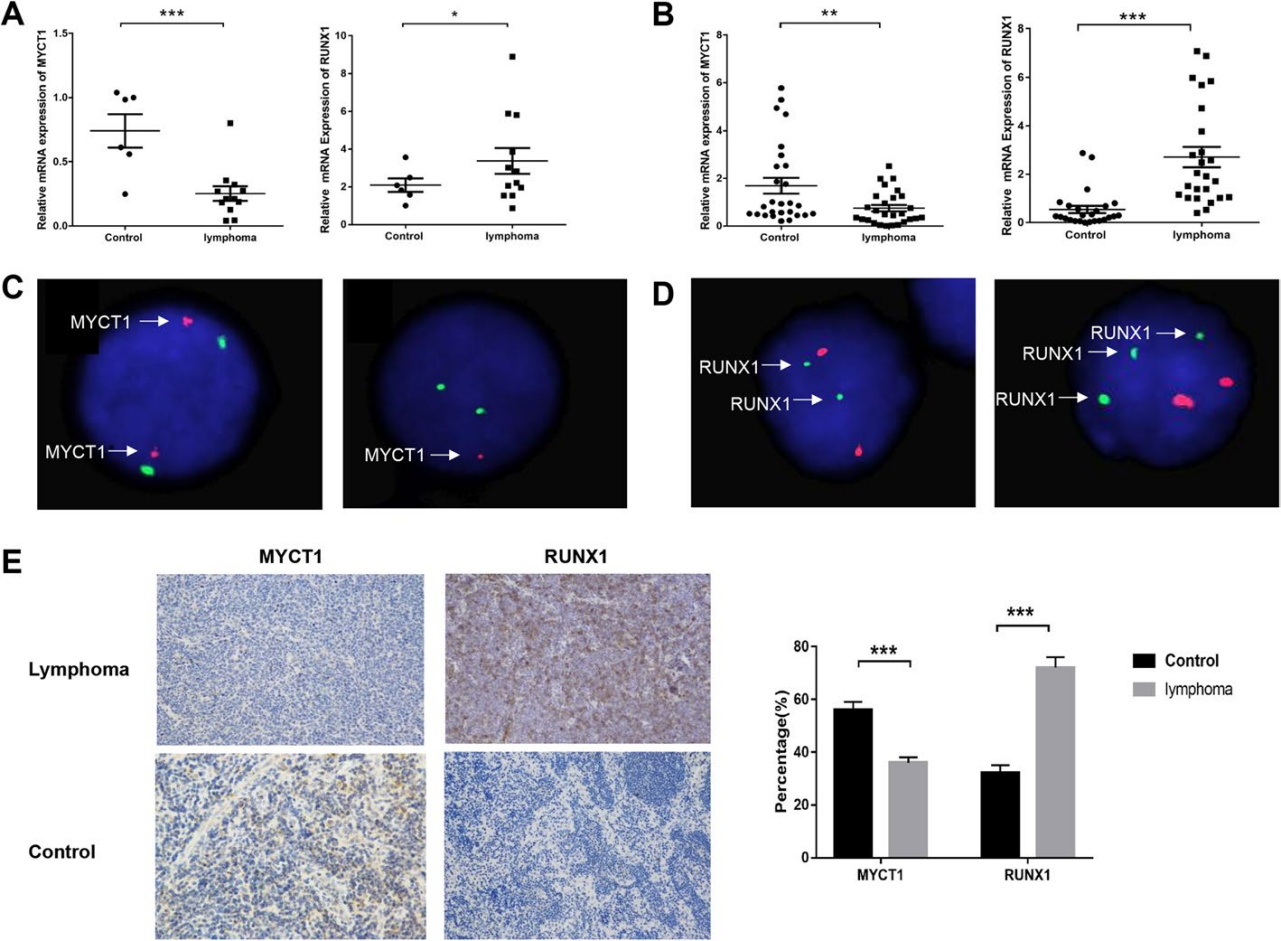
Detection of MYCT1 and RUNX1 mRNA, DNA and protein in lymphoma patients and the control group. **A** MYCT1 and RUNX1 mRNA expression in lymphoma patients with bone marrow infiltration and normal bone marrow control group. **B** MYCT1 and RUNX1 mRNA expression in paraffin-embedded tissues of the lymphoma and reactive lymphadenitis control group. **C** Representative MYCT1 FISH test results. The left figure shows the FISH results with a normal copy number of MYCT1; the figure on the right shows the FISH results of MYCT1 copy number deletion. The red signal represents the chromosome 6q25 region of the MYCT1 gene, and the green signal represents the chromosome 6p11.1-q11.11 region of the CEP6 gene. **D** Representative RUNX1 FISH test results. The left figure shows the FISH results with a normal RUNX1 copy number; the right figure shows the FISH results of RUNX1 copy number amplification. The red signal represents the chromosome 8q21 region, where the ETO gene is located, and the green signal represents the chromosome 21q22 region, where the RUNX1 gene is located. **E** MYCT1 and RUNX1 protein expression in paraffin-embedded tissues of the lymphoma and reactive lymphadenitis control group (IHC × 200) detected by immunohistochemical DAB staining. The data represent the results of three independent repeated experiments. *, * * and * * * represent *p* < 0.05, *p* < 0.01 and *p* < 0.001, respectively

The expression levels of *MYCT1* and *RUNX1* mRNA in the bone marrow of 12 lymphoma patients with bone marrow infiltration were detected by qPCR. The results showed that compared with the normal bone marrow control group, the mRNA expression levels of MYCT1 and RUNX1 in the bone marrow of lymphoma patients were significantly lower and higher, respectively (Fig. 3A).

The expression levels of MYCT1 and RUNX1 mRNA in paraffin-embedded tissues of 27 cases of lymphoma were detected by qPCR. The results showed that compared with the reactive lymphadenitis control group, the mRNA expression levels of MYCT1 and RUNX1 in lymphoma paraffin-embedded tissues were significantly lower and higher, respectively (Fig. 3B).

MYCT1 and RUNX1 site-specific FISH probes were used to detect the copy number of MYCT1 and RUNX1 DNA in bone marrow samples of 78 patients with malignant lymphoma with abnormal karyotypes. The results showed that 20 patients had MYCT1 deletion, accounting for 25.6% (Fig. 3C); RUNX1 amplification was present in 16 patients, with an amplification rate of 20.5% (Fig. 3D); and five patients had MYCT1 deletion and RUNX1 amplification at the same time, accounting for 5.4% of the total (Additional file 1: Table S2). The above results showed that there was a negative correlation between MYCT1 gene expression and RUNX1 gene expression in lymphoma cells with bone marrow infiltration.

Immunohistochemical results showed that MYCT1 was expressed in the nucleus and cytoplasm of lymphoma, mainly in the nucleus, and was expressed at low levels and was positive. MYCT1 was positive in 10 of 27 patients with lymphoma, with a positive rate of 37%. MYCT1 was positive in 15 of 27 patients with reactive lymphadenitis, with a positive rate of 56%. RUNX1 protein was localized in the cytoplasm, and most of the values were moderately or highly expressed and positive. Among 27 patients with lymphoma, 19 cases were RUNX1 positive, with a positive rate of 70%. Among 27 patients with reactive lymphadenitis, 9 cases were positive for RUNX1, with a positive rate of 33% (Fig. 3E).

**Fig. 4:**
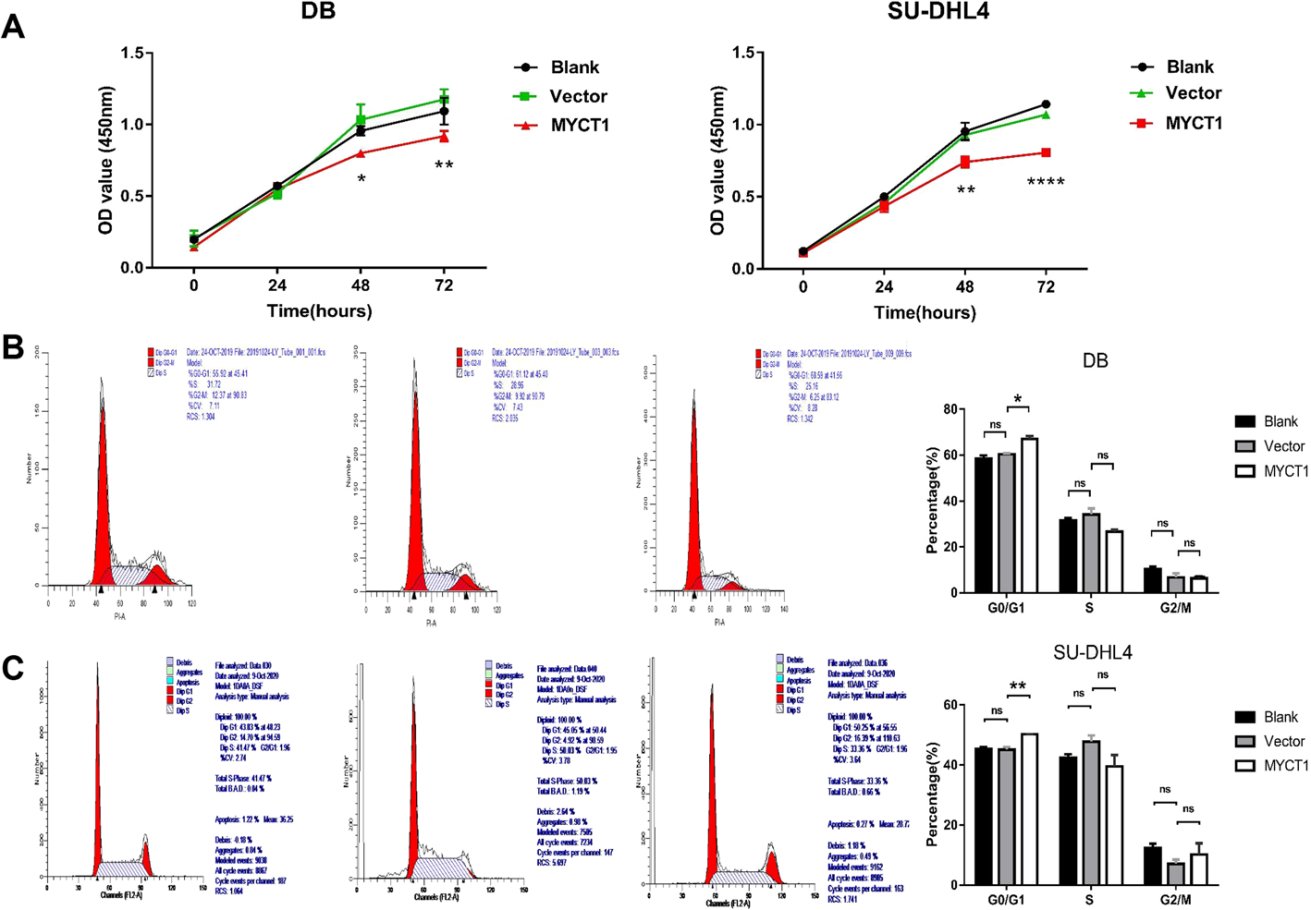
Effects of MYCT1 on the proliferation and cell cycle of the DLBCL cell lines DB and SU-DHL4. **A** Effect of MYCT1 on the proliferation of DB and SU-DHL4 cell lines. The left figure shows the effect of MYCT1 on the proliferation of DB cells; the right figure shows the effect of MYCT1 on the proliferation of SU-DHL4 cells. **B** Effect of MYCT1 on the cell cycle of DB cells. **C** Effect of MYCT1 on the cycle of the SU-DHL4 cell line. The data represent the results of three independent repeated experiments. *, * * and * * * represent *p* < 0.05, *p* < 0.01 and *p* < 0.001, respectively, and NS represents no statistically significant difference

In the study of DLBCL cell lines DB and SU-DHL4, we found that in the CCK8 test, after 24 h of transfection, the proliferation ability of the MYCT1 overexpression group, control group and blank control group was not significantly abnormal, but at 48 h, the proliferation ability of the MYCT1 overexpression stable cell line was significantly reduced (DB group *p* < 0.05, SU-DHL4 group *p* < 0.01). At 72 h, the reduction efficiency was more obvious (*p* < 0.01 in the DB group and *p* < 0.001 in the SU-DHL4 group) (Fig. 4A). The above results suggest that MYCT1 overexpression can significantly inhibit the proliferation of DLBCL cells. The results of cell cycle detection showed that compared with the control group, the proportion of DB and SU-DHL4 cell lines stably transformed with MYCT1 overexpression vector in G0/G1 phase increased significantly (*p* < 0.05), while there was no significant difference between S phase and G2/M phase (Figs. 4B, 3C), suggesting that MYCT1 can regulate the DLBCL cell cycle and block it in G0/G1 phase.

**Fig. 5:**
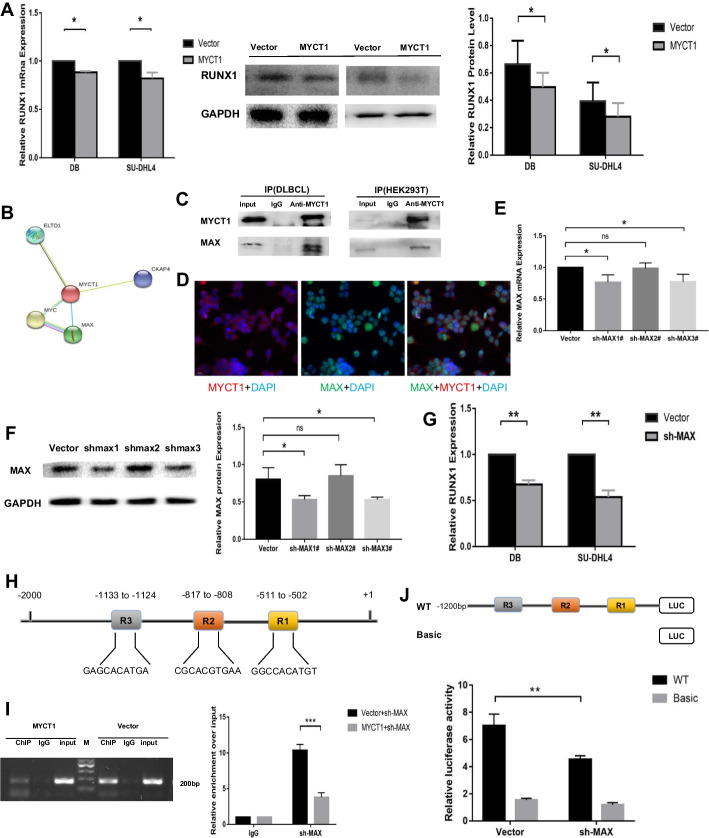
The regulatory mechanism of RUNX1 by MYCT1. **A** Effect of MYCT1 on the expression level of RUNX1 in the DLBCL cell lines DB and SU-DHL4. The left figure shows the effect of MYCT1 overexpression on the mRNA expression level of RUNX1 in DB and SU-DHL4 cells; the right figure shows the effect of MYCT1 overexpression on the protein expression level of RUNX1 in DB and SU-DHL4 cells. **B** Interaction diagram of MYCT1 and MAX predicted by STRING bioinformatics software. **C** Test results of binding ability between MYCT1 and MAX protein in DLBCL and HEK293T cells. The upper figure shows the application of anti-MAX antibody for immunoprecipitation. The lower figure shows the application of anti-MYCT1 antibody for immunoprecipitation. **D** The subcellular location of MYCT1 and MAX protein in HEK293T cells by immunofluorescence staining. **E** Detection results of MAX knockdown efficiency in DLBCL (mRNA level). **F** Detection results of MAX knockdown efficiency in DLBCL (protein level). **G** Effect of MAX knockdown on RUNX1 expression in DLBCL. **H** Schematic diagram of potential MAX binding sites in the RUNX1 promoter region. **I** ChIP detect results showed MYCT1 inhibits the binding of MAX to the RUNX1 promoter region. **J** Schematic diagram of the luciferase truncated vector containing MAX binding sites in the RUNX1 promoter region and the effect of MAX knockdown on the transcriptional activity of the RUNX1 promoter region in DLBCL cells. Data represent the results of three independent repeated experiments. *, * * and * * * represent *p* < 0.05, *p* < 0.01, and *p* < 0.001, respectively; NS represents no statistical significance

Real-time PCR and Western blotting were used to detect the expression of RUNX1 after stable transformation of the MYCT1 overexpression vector in the DLBCL cell lines DB and SU-DHL4. The results showed that the expression levels of RUNX1 mRNA and protein after MYCT1 overexpression were significantly lower than those in the control group (*p* < 0.05) (Fig. 5A). The above results are consistent with the results of previous clinical samples, suggesting that MYCT1 plays a negative role in regulating the expression of RUNX1 in DLBCL cells.

The predicted results of STRING bioinformatics software showed that MAX interacts with MYCT1 (Fig. 5B). The results of forward and reverse Co-IP experiments showed that MYCT1 can specifically bind to MAX in DLBCL and HEK293T cells (Fig. 5C). Immunofluorescence assays results showed that MYCT1 expressed in both cytoplasm and nucleus while MAX in the nucleus mainly, and they co-located in nucleus (Fig. 5D).

We synthesized three sh-MAX RNAs—sh-MAX1#1, sh-MAX1#2 and sh-MAX1#3—and transfected them into DLBCL cell lines. The results showed that the mRNA and protein levels of MAX in the sh-MAX1#1 and sh-MAX1#3 transfection groups were significantly reduced, suggesting that sh-MAX1#1 and sh-MAX1#3 were knocked down successfully (*p* < 0.05) (Fig. 5E, F). We chose sh-MAX1#3 for subsequent experiments. We also detected the expression level of RUNX1 in DLBCL cell lines with MAX knockdown by real-time PCR. The results showed that the expression level of RUNX1 decreased significantly, suggesting that MAX can positively regulate the expression level of RUNX1 in DLBCL cells (*p* < 0.05) (Fig. 5G).

Jaspar bioinformatics software predicted that there were multiple potential MAX binding sites in the RUNX1 promoter region; − 1133 to − 1124 bp, − 817 to − 808 bp and − 511 to − 502 bp were three binding sites with a binding threshold score of more than 90%. Therefore, we selected these three sites for the follow-up study (Fig. 5H).

To further study the mechanism by which MYCT1 regulates RUNX1, we detected the effect of MYCT1 on the binding ability of MAX to the RUNX1 promoter region by chromatin immunoprecipitation combined with PCR. The results showed that the protein/DNA complex precipitated by the anti-MAX antibody could be amplified by using RUNX1 promoter region-specific primers, and the amount of RUNX1-PCR products in MYCT1 stable cells was lower than that in the empty body group (*p* < 0.001) (Fig. 5I). These findings suggest that in DLBCL cells, MYCT1 inhibits the binding ability of the latter to the RUNX1 promoter region by binding to MAX, thereby inhibiting the expression of RUNX1.

We constructed a luciferase reporter gene expression vector (Fig. 5J) of − 2000 bp upstream of the RUNX1 promoter region and cotransfected it with sh-MAX into HEK293T cells, further verifying the binding ability of MAX and the RUNX1 promoter region in vitro. The results showed that MAX significantly promoted the transcriptional activity of the RUNX1 promoter region, while knockdown of MAX significantly inhibited the transcriptional activity of the RUNX1 promoter region (*p* < 0.01) (Fig. 5J).

### MYCT1 inhibits proliferation in diffuse large B-cell lymphoma probably by suppressing RUNX1 transcription

**Fig. 6:**
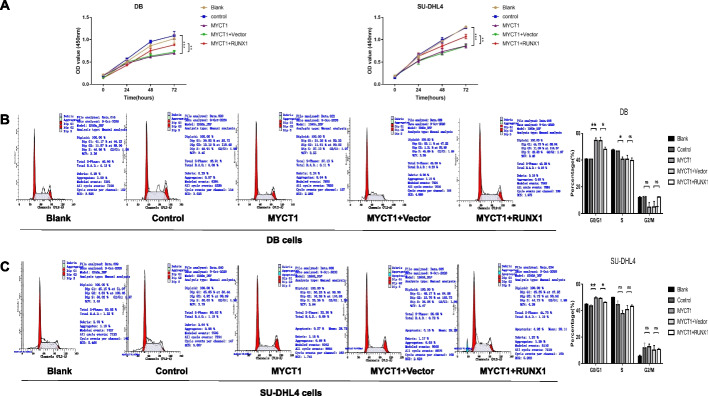
RUNX1 restored MYCT1 in the proliferation and cell cycle of DLBCL cells. **A** RUNX1 restored MYCT1 in the proliferation of DB and SU-DHL4 cells. **B** RUNX1 responded to the effect of MYCT1 on the DB cycle. **C** RUNX1 responded to the effect of MYCT1 on the SU-DHL4 cycle. *, * *, and * * * represent *p* < 0.05, *p* < 0.01 and *p* < 0.001, respectively, and NS represents no significant difference

To investigate whether MYCT1 affects the proliferation and cell cycle progression of DLBCL cells through RUNX1, we used CCK8 and flow cytometry to detect whether overexpression of RUNX1 can restore the effects of MYCT1 on the proliferation and cell cycle progression of DLBCL cells. The results showed that RUNX1 significantly restored the inhibitory effect of MYCT1 on the proliferation of DLBCL cells and the promotion of DLBCL cell cycle arrest (Fig. 6A–D). These results suggest that MYCT1 inhibits the proliferation of DLBCL cells through RUNX1.

In conclusion, our results suggest that MYCT1 inhibits the expression of RUNX1 by inhibiting the binding ability between MAX and the RUNX1 promoter region, thereby affecting the proliferation and cell cycle process of DLBCL cells.

## Discussion

Chromosomes are mainly composed of highly compacted spiral DNA, which is the carrier of genes. Chromosomal instability (CIN) is caused by incorrect separation of somatic cells during mitosis, which can manifest as numerical and structural aberrations and is a notable feature of tumors [17]. The change in chromosome number is known as aneuploidy, that is, the loss or acquisition of the whole chromosome. Chromosomal abnormalities are characteristics of human tumors and are found in almost all solid tumors and malignant hematological diseases. Changes in chromosome structure are mainly the loss of chromosome heterozygosity, chromosome translocation, insertion translocation, inversion and amplification caused by chromosome breakage and error repair. Since Lengauer [18] and colleagues found the role of CIN in human tumors, an increasing number of researchers have been paying attention to the mechanism of CIN and its relationship with human malignant tumors.

The replication of DNA is a highly regulated process. If the cell cycle is blocked, genetic material will replicate and separate abnormally [19]. In the whole process of the cell cycle, exogenous and endogenous stress responses can lead to abnormal DNA synthesis and damage repair [20]. However, epigenetic abnormalities, such as telomeres and centromeres, can lead to abnormalities in the repetitive sequence of chromosome secondary structure, chromatin conformation, origin and distribution, and replication time, resulting in replication-related DNA double-strand breaks, increased chromosome instability, and thus karyotype abnormalities [21].

Lymphoma is a highly heterogeneous disease. Ninety percent of lymphoma patients have clonal chromosomal abnormalities, with a large number of gene mutations, chromosome number changes and structural abnormalities [22]. DLBCL tumor cells usually have random and complex chromosomal abnormalities and sometimes show more than two kinds of chromosomal abnormal variations, which indicates that DLBCL patients have karyotype genetic instability and may undergo additional genetic changes [23].

In 1972, Manolov [24] first found that 14q + was closely related to Burkitt lymphoma. Later, researchers turned their research focus to lymphoma cytogenetics. Studies have shown that chromosomal reproducibility and clonal abnormalities have greatly affected the classification and subtype diagnosis of NHLs. Specific marker chromosomes of various lymphomas have been found successively, such as t (14;18) in follicular lymphoma, t (8;14) in Burkitt lymphoma, t (3;14) in diffuse large B-cell lymphoma, t (11;14) in mantle cell lymphoma, and t (2;5) in anaplastic large cell lymphoma and their derived abnormalities [25, 26].

In this study, we found that the overall abnormal rate of karyotypes in lymphoma patients with bone marrow infiltration can reach 37.3%, in which the abnormal rate of complex karyotypes can be as high as 87.2%, which is consistent with the research results of scholars such as Mertens [27]. In addition, we found that the platelet value of patients with abnormal karyotypes was relatively low at the initial diagnosis; the complete remission rate, OS, and PFS were significantly reduced; and the three-year mortality was significantly increased. These findings are consistent with Greenwell [28], but the regulatory mechanism between complex karyotype abnormalities and thrombocytopenia has not been reported.

The smallest overlapping regions of chromosome deletion are important evidence for the discovery of tumor suppressor genes [29]. In this study, we found the three smallest overlapping regions in lymphoma patients with bone marrow infiltration, namely, 14q32-qter, 6q21-25, and 11q23-qter. Patients with abnormalities in these three key chromosomal regions have a shortened trend in OS and PFS. It can be seen that patients with abnormalities in these smallest overlapping regions are prone to rapid disease progression and die in the early stage; thus, the disease is more dangerous in these patients. These results suggest that there may be tumor suppressor genes related to the occurrence, development and prognosis of lymphoma in these regions.

Some scholars have also studied these three smallest overlap regions. In a study of the correlation between the pathogenesis of lymphoma and disease subtypes, Lossos [30] and others found that the rearrangement of the IGH gene at the 14q32 site, such as t (14;18), t (11;14), t (8;14) and other initial genetic changes, is related to the occurrence of lymphoma. 11q23.1 is an unstable region of B-cell lymphoma [31]. The FOXR1 gene and KMT2A in the 11q23 segment are related to the occurrence of B-cell lymphoma [32, 33]. 6q21-q5 is also an important chromosomal region associated with lymphoma. Abnormalities at different sites of 6q can affect the malignancy of lymphoma; for example, 6q21 is related to high-grade lymphoma, and 6q23 is related to low-grade lymphoma [34]. Some scholars have found in T-cell lymphoma that the deletion of the 6q25 chromosome segment is related to the prognosis of lymphoma [35], but these studies involve large chromosome segments and many genes. To date, relevant research reports on specific genes in this segment are rare.

Previously, we showed that MYCT1, the first tumor suppressor gene cloned in our laboratory, plays an important role in the occurrence and development of many kinds of tumors. Moreover, MYCT1 is located in the region of chromosome 6q25. At present, there has been no report on whether MYCT1 can affect the stability of chromosome karyotypes or its relationship with the occurrence and development of lymphoma.

The study of clinical specimens has suggested that MYCT1 expression is reduced in lymphoma and thus may play a role as a tumor suppressor gene in lymphoma. We selected two DLBCL cell lines for culture and karyotype analysis. The DB cell line has a super triploid karyotype, and the SU-DHL4 cell line has a near-diploid karyotype, both of which are complex karyotypes. The analysis showed that both cell primordial karyotypes contained the t(14;18) translocation and involved multiple chromosomal structural and numerical abnormalities. When the MYCT1 stable cell line was successfully constructed, we analyzed the karyotypes of the two cell lines again and found that both cell lines had karyotype evolution and structural abnormalities. In the DB-MYCT1 group, the t (2;8) translocation occurred, and an abnormal chromosome with a long arm of chromosome 7 was added; the SU-DHL4-MYCT1 group showed deletion of the long arm of chromosome 7 and abnormalities of the short arm of chromosomes 9, 18 and 22. MYCT1 has an impact on the stability of DLBCL chromosomes, resulting in the increase or deletion of large segments of chromosomes.

In this study, the proliferation and cycle changes of DLBCL cells stably transformed with MYCT1 were studied. MYCT1 overexpression reduced the proliferation of DLBCL cells and blocked the cells in G0/G1, which played a significant negative regulatory role in DLBCL cells, once again confirming the role of MYCT1 as a tumor suppressor gene in lymphoma.

Studies have found that when some tumor suppressor genes are silenced in cells with stable chromosomes, replication stress increases the number of structural chromosomal aberrations [36]. The two-way interaction between replication stress and chromosomal error segregation has changed chromosomal instability, providing an evolutionary mechanism for cancer cells [37]. Other scholars have found that tumor cells with abnormal karyotypes have evolved a mechanism to escape the immune system, and changing CIN can regulate tumor activity and immunogenicity [38]. In summary, we consider that after MYCT1 overexpression, it may interact with some transcription factors or proteins, causing cell cycle arrest, inactivating oncogenes or activating tumor suppressor genes of the DLBCL cell line itself, and losing the previously stable immune escape function, which will lead to increased apoptosis and inhibit the proliferation of lymphoma cells.

The coding product of the MAX gene is MYC-associated factor X (MAX), which is a highly conserved transcription factor highly homologous to the primary structure of C-MYC. MAX can regulate the transcription of target genes and regulate cell proliferation, apoptosis and differentiation [39]. Moreover, MAX is the core component of the C-MYC regulatory transcription complex and is a necessary factor for C-MYC to bind DNA and activate transcription [40].

RUNX1 is a key regulator in hematopoiesis, a common target of multiple chromosome translocations in human leukemia, and it plays an important role in hematopoiesis regulation and the occurrence and development of hematological malignancies [41, 42]. Some scholars have found that combined transgenic mice, with T cells or B cells overexpressing MYC and RUNX1 genes, are easily accessible to lymphoma, suggesting that RUNX1 can accelerate MYC-induced lymphoma [43]. The RUNX1 gene can also be used as a target for mouse leukemia virus (MLV) insertion mutation and lymphoma transcription activation [44]. In contrast, the cells of RUNX1-deficient chimeric mice can also develop T-cell lymphoma after treatment with ENU, suggesting that the loss of RUNX1 activity may also lead to lymphoid malignancies [45]. Borland [46] also found that RUNX1 deficiency can cause lymphoma and proposed that RUNX1 can be used as a therapeutic target in p53 wild-type or mutant lymphoma. An increasing number of research results have suggested that the combination of positive or negative regulators and RUNX1 may be related to their functions in tumors [47].

In a study of RUNX1 regulating cell proliferation and apoptosis, scholars found that RUNX1 can upregulate centromere-associated protein E (CENPE), lead to the early expression of genes involved in the cell cycle and repeated application, and promote the growth of AML cells through cell proliferation [48]. Jenkins [49] found that RUNX1 upregulates the expression of type 1 insulin-like growth factor receptor (IGF1R), thereby inhibiting T-ALL cell apoptosis and promoting proliferation. Martinez-Soria [50] found in a study of cell cycle regulation that RUNX1 mutation in AML can activate the transcription of CCDN2 together with AP-1 and then block the cell in G1 phase. In lymphoma, the mechanism of RUNX1 and cell cycle regulation has not been reported.

Besides, RUNX1 also plays an important roles in solid tumors metastasis such as colorectal cancer, lung cancer and glioblastoma [51–53]. As reported, Runx1 also participates in glucocorticoid resistance in lymphomagenesis [54]. Therefore, effects of MYCT1 on migration and drug resistance of lymphoma cells needs further study.

In this study, we found that (1) MYCT1 is located in one of the three smallest overlapping regions of diffuse large B-cell lymphoma; (2) MYCT1 alters the chromosomal instability of diffuse large B-cell lymphoma cells; (3) MYCT1 is negatively correlated with RUNX1 in lymphoma patients and MYCT1 represses RUNX1 transcription by binding MAX in diffuse large B-cell lymphoma cells; and (4) MYCT1 inhibits proliferation in diffuse large B-cell lymphoma probably by suppressing RUNX1 transcription.

In conclusion, MYCT1 overexpression can inhibit the positive regulation of RUNX1 by MAX, resulting in the downregulation of RUNX1 expression. Through a series of experiments, we proved the regulation of the MYCT1-MAX-RUNX1 signaling pathway in DLBCL cells and confirmed that MYCT1 plays the role of its tumor suppressor gene in lymphoma. This experiment is the first to study the function and mechanism of MYCT1 in lymphoma, which provides a new target for further study of the pathogenesis and early diagnosis and treatment of lymphoma.

## Conclusions

In this study, we found MYCT1 could inhibit proliferation and promote cell cycle arrest in diffuse large B-cell lymphoma cells. Moreover, we demonstrate that MYCT1 represses RUNX1 transcription by binding MAX. The findings provide clues and a basis for in-depth studies of MYCT1 in the diagnosis, treatment and prognosis of lymphoma.

### Supplementary Information

**Additional file 1: Table S1.** chromosome karyotype number and structural aberrations in patients with abnormal karyotype. **Table S2.** MYCT1 and RUNX1 FISH test results.

## Data Availability

The dataset used and analyzed during the current study is available from the corresponding author on reasonable request.

## Abbreviations

HL: Hodgkin lymphoma
NHL: Non-Hodgkin lymphoma
DLBCL: Diffuse large B-cell lymphoma
MYCT1: MYC target 1
CIN: Chromosomal instability
RUNX1: Runt related protein 1
MAX: MYC associated factor X
OS: Overall survival
PFS: Progression-free survival
FISH: Fluorescence in situ hybridization
Co-IP: Coimmunoprecipitation
ChIp: Chromatin immunoprecipitation
