# Supplementary material for: MYCT1 inhibits hematopoiesis in diffuse large B-cell lymphoma by suppressing RUNX1 transcription
Source: Cell Mol Biol Lett. 2024 Jan 3;29:5. doi: 10.1186/s11658-023-00522-0 (PMC10763471; doi:10.1186/s11658-023-00522-0)
Supplement: Supplementary file 1 — Additional file 1: Table S1. chromosome karyotype number and structural aberrations in patients with abnormal karyotype. Table S2. MYCT1 and RUNX1 FISH test results. [file 11658_2023_522_MOESM1_ESM.docx]

**Supplementary Table 1 chromosome karyotype number and structural aberrations in patients with abnormal karyotype**

| chromosome | number（%） | | structural（%） | chromosome | Number （%） | | structural（%） |
| --- | --- | --- | --- | --- | --- | --- | --- |
|  | Amplification Deletion | |  |  | Amplification Deletion | |  |
| X | 3(0.95%) | 4(0.68%) | 3(1.10%) | 11 | 7(2.21%) | 6(1.89%) | 28(10.3%) |
| Y | 2(0.63%) | 7(2.21%) | 1(0.37%) | 12 | 7(2.21%) | 6(1.89%) | 4(1.47%) |
| 1 | 2(0.63%) | 5(1.58%) | 20(7.35%) | 13 | 5(1.58%) | 9(2.84%) | 10(3.68%) |
| 2 | 5(1.58%) | 3(0.95%) | 15(5.51%) | 14 | 9(2.84%) | 5(1.58%) | 23(8.46%) |
| 3 | 13(4.10%) | 10(3.15%) | 19(6.99%) | 15 | 4(1.26%) | 9(2.84%) | 2(0.74%) |
| 4 | 2(0.63%) | 10(3.15%) | 13(4.78%) | 16 | 5(1.58%) | 9(2.84%) | 2(0.74%) |
| 5 | 7(2.21%) | 5(1.58%) | 13(4.78%) | 17 | 7(2.21%) | 11(3.47%) | 12(4.41%) |
| 6 | 4(1.26%) | 7(2.21%) | 34(12.5%) | 18 | 9(2.84%) | 10(3.15%) | 4(1.47%) |
| 7 | 6(1.89%) | 7(2.21%) | 13(4.78%) | 19 | 3(0.95%) | 9(2.84%) | 3(1.10%) |
| 8 | 9(2.84%) | 10(3.15%) | 15(5.51%) | 20 | 5(1.58%) | 6(1.89%) | 3(1.10%) |
| 9 | 4(1.26%) | 8(2.52%) | 15(5.51%) | 21 | 10(3.15%) | 5(1.58%) | 7(2.57%) |
| 10 | 5(1.58%) | 11(3.47%) | 4(1.47%) | 22 | 7(2.21%) | 4(1.26%) | 9(3.31%) |

**Supplementary Table 2 MYCT1 and RUNX1 fish test results**

| Number | MYCT1 Deletion | RUNX1 Amplification | Number | MYCT1 Deletion | RUNX1 Amplification | Number | MYCT1 Deletion | RUNX1 Amplification |
| --- | --- | --- | --- | --- | --- | --- | --- | --- |
| 1 | （+） | （+） | 27 | （-） | （-） | 53 | （+） | （-） |
| 2 | （-） | （-） | 28 | （-） | （-） | 54 | （-） | （-） |
| 3 | （+） | （+） | 29 | （-） | （+） | 55 | （-） | （-） |
| 4 | （-） | （-） | 30 | （-） | （+） | 56 | （+） | （-） |
| 5 | （+） | （-） | 31 | （-） | （+） | 57 | （-） | （-） |
| 6 | （+） | （+） | 32 | （-） | （-） | 58 | （-） | （-） |
| 7 | （+） | （-） | 33 | （-） | （-） | 59 | （-） | （-） |
| 8 | （-） | （-） | 34 | （+） | （-） | 60 | （-） | （-） |
| 9 | （+） | （-） | 35 | （-） | （-） | 61 | （-） | （-） |
| 10 | （-） | （+） | 36 | （-） | （-） | 62 | （-） | （+） |
| 11 | （-） | （-） | 37 | （-） | （-） | 63 | （-） | （-） |
| 12 | （-） | （+） | 38 | （+） | （-） | 64 | （-） | （-） |
| 13 | （+） | （+） | 39 | （+） | （-） | 65 | （-） | （-） |
| 14 | （+） | （+） | 40 | （-） | （-） | 66 | （-） | （+） |
| 15 | （-） | （-） | 41 | （-） | （-） | 67 | （-） | （-） |
| 16 | （-） | （-） | 42 | （-） | （-） | 68 | （-） | （-） |
| 17 | （-） | （+） | 43 | （-） | （+） | 69 | （-） | （+） |
| 18 | （-） | （-） | 44 | （-） | （-） | 70 | （-） | （-） |
| 19 | （+） | （-） | 45 | （-） | （+） | 71 | （-） | （-） |
| 20 | （-） | （-） | 46 | （-） | （-） | 72 | （-） | （-） |
| 21 | （-） | （-） | 47 | （-） | （-） | 73 | （-） | （-） |
| 22 | （+） | （-） | 48 | （-） | （-） | 74 | （+） | （-） |
| 23 | （+） | （-） | 49 | （-） | （-） | 75 | （-） | （-） |
| 24 | （+） | （-） | 50 | （+） | （-） | 76 | （-） | （-） |
| 25 | （-） | （+） | 51 | （+） | （-） | 77 | （-） | （-） |
| 26 | （-） | （+） | 52 | （-） | （-） | 78 | （-） | （-） |
